# Real-Time Monitoring of the Position and Orientation of a Radio Telescope Sub-Reflector with Fiber Bragg Grating Sensors

**DOI:** 10.3390/s19030619

**Published:** 2019-02-01

**Authors:** Yong Zhao, Jingli Du, Qian Xu, Hong Bao

**Affiliations:** 1 Key Laboratory of Electronic Equipment Structure Design of Ministry of Education, Xidian University, Xi’an 710071, China; henanzy1984@163.com (Y.Z.); jldu@mail.xidian.edu.cn (J.D.); 2 Xinjiang Observatory, National Astronomical Observatories, Chinese Academy of Sciences, Urumqi 830011, China; xuqian@xao.ac.cn

**Keywords:** environmental loads, sub-reflector shift, supporting structure, inverse finite element method, FBG strain sensors

## Abstract

Environmental loads linked with pointing errors, such as gravity, thermal gradients, and wind disturbances, are a serious concern for large-aperture high-frequency radio telescopes. For the purpose of maintaining the pointing performance of a telescope, a contact measurement scheme is proposed on basis of fiber Bragg grating (FBG) strain sensors that can monitor the sub-reflector shift in real time as the input data of the adjustment system. In this scheme, the relationship between the in situ strain measurement and the deformation of the supporting structure, which is the main cause of sub-reflector shift, is deduced using the inverse Finite Element Method (iFEM). Finally, experimental studies are carried out on a simple physical structure model to validate the effectiveness and accuracy of the contact measurement scheme.

## 1. Introduction

With the aim of satisfying the pointing performance of a large-aperture high-frequency radio telescope, it is important to maintain the ideal location relationship between the sub-reflector and the main reflector. Nevertheless, environmental loads such as gravity, thermal gradients, and wind disturbances may deform the shape of the supporting structure of the sub-reflector, which will seriously affect the location relationship.

In a series of three papers, the active control system has been widely used for adjusting the position and orientation of the sub-reflector [1,2,3]. In order to provide the exact input for the active control system, the position and orientation of the sub-reflector must be firstly and accurately obtained. For this purpose, a non-contact measurement scheme based on a Position Sensing Device (PSD) consisting of a laser diode and a Complementary Metal Oxide Semiconductor (CMOS) camera was developed by the Sardinia Radio Telescope (SRT) Metrology team [4,5]. In this scheme, the accuracy of the PSD was able to reach around 0.1 mm within the measurement range of ±40 mm. Because the laser-catching capability of the CMOS camera is seriously affected by ambient light, it is only during the night that this non-contact measurement scheme can be successfully performed.

To overcome the aforementioned limitation, a contact measurement technology based on sensing structural deformations from in situ strain measurements, namely shape sensing, has offered a method for structural health monitoring (SHM) that can be applied to active control systems of large structures [6,7,8]. Importantly, compared to traditional strain sensors, fiber Bragg grating (FBG) strain sensors have been extensively studied and applied for shape sensing due to their lightness, accuracy, and resistance to electromagnetic interference, radio frequency interference, and radiation [9,10,11]. 

**Figure 1:**
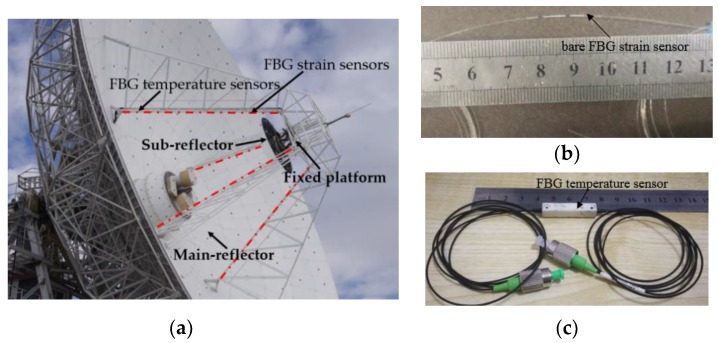
(**a**) Radio telescope and sensor placement, (**b**) bare fiber Bragg grating (FBG) strain sensor, and (**c**) FBG temperature sensor.

For a typical radio telescope, the supporting structure comprises a fixed platform and four supporting legs; the sub-reflector is fixed on the fixed platform (Figure 1a). Neglecting the deformation of the fixed platform (the stiffness of the fixed platform is big enough to resist environmental loads), the sub-reflector shift mainly arises from the fixed platform shift associated with the deformations of the four supporting legs caused by the weight of the sub-reflector. Thus, when the end-node shifts of the inside beams of the four supporting legs are known, the position of the fixed platform can be determined. Also, the orientation of the fixed platform can be computed from the end-node shifts with a direction cosine algorithm. Furthermore, the position and orientation of the sub-reflector can be adjusted using the Stewart platform attached to the fixed platform (Appendix A).

Therefore, the present paper aims to develop a contact measurement scheme according to shape sensing technology and bare FBG strain sensors (Figure 1b) in order to measure the deformations of the inside beams of the four supporting legs in real time. The key of this scheme is to construct a suitable and robust measurement model to describe the relationship between the beam deformations and the in situ strain measurements. Also, in order to monitor the influence of the temperature variation on the strain measurement system, several FBG temperature sensors (Figure 1c) are set on the surface of the beam used to capture the current temperature (Figure 1a). When the current temperature is obtained, the wavelength shift of the FBG strain sensor caused by temperature variation can be estimated, and then this wavelength shift can be removed from the strain measurement. 

In order to sense the deformation of the beam element, a load-independent method was developed by Ko et al. according to the piece-wise continuous polynomials and the classical beam theory [12,13]. This one-dimensional scheme has been demonstrated to be sufficiently accurate for predicting deformation and somewhat less accurate for evaluating the cross-sectional twist of a beam/frame structure; however, a large number of strain sensors are required to capture in situ strain data. Bogert et al. analyzed and verified the effectiveness of the modal transformation method for reconstructing the structural deformation [14]. Despite the advantages of this method, a great many natural vibration modes are required. Another problem is that this method requires an analysis of numerous eigenvalues and a detailed description of the elastic and inertial material properties. Therefore, a suitable algorithm for shape sensing should be [15]: (1) general enough to accommodate complex structural topologies and boundary conditions; (2) robust, stable, and accurate under a wide range of loads, material systems, inertial/damping characteristics, and inherent errors during the strain measurements; (3) sufficiently fast for applications in real time.

An algorithm framework which can fulfill the aforementioned requirements was proposed by Tessler and Spangler [16]. This algorithm, named the inverse Finite Element Method (iFEM), employs the weighted least squares variational principle to construct a moderate relationship between the strain measurements and the deformations of a structure such as a frame (truss and beam), plate, and shell, etc. On the basis of the iFEM framework and the kinematic assumptions of Timoshenko beam theory, Gherlone et al. formulated a robust inverse finite beam element for the purpose of sensing three-dimensional (3D) deformation of a beam/frame structure [15,17]. In References [18,19], the optimal placement of sensors was researched by Zhao et al. to maintain the stability and accuracy of deformation reconstruction using iFEM with an inverse beam element. The experimental validations showed that iFEM is able to accurately and effectively estimate the deformations of a three-dimensional frame/shell/plate, and even the composite structure undergoing static and/or dynamic damped harmonic excitations without any knowledge of material, inertial, loading, or damping structural properties [20]. 

**Figure 2:**
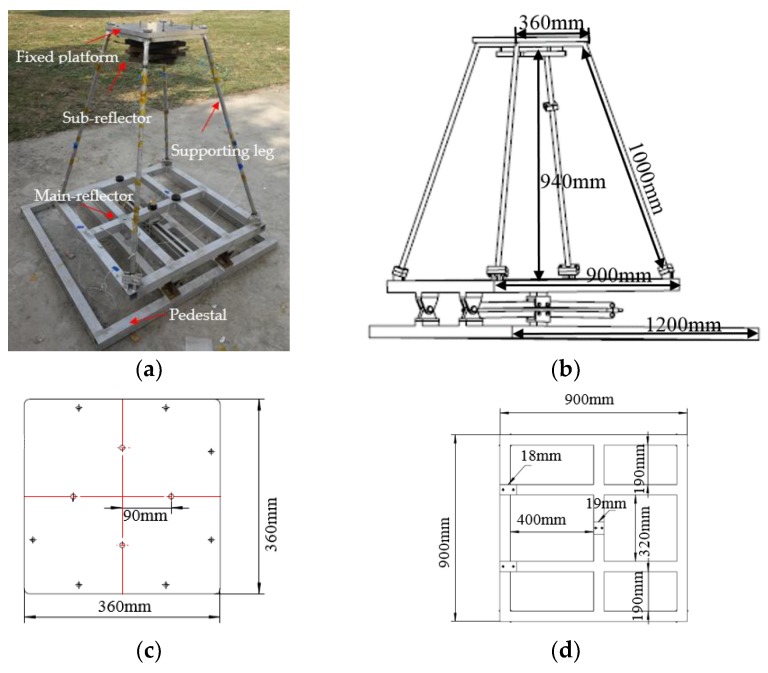

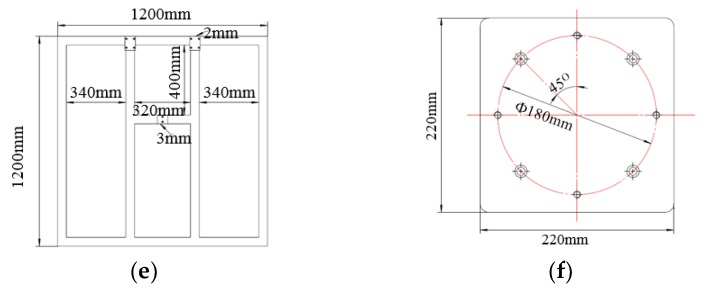
The simple physical model of the radio telescope and its size: (**a**) Telescope model; (**b**) The size of the whole structure; (**c**) The size of the fixed platform; (**d**) The size of the main reflector; (**e**) The size of the pedestal; (**f**) The size of the iron plate.

According to the advantages of the method described above, iFEM is employed to derive the measurement model for the description of the relationship between the beam deformations (the end-node shifts of the four inside beams) and the in situ strain measurements in the present paper. To verify the effectiveness and feasibility of the measurement scheme, a simple physical structure capable of modeling the whole telescope was manufactured (Figure 2a). The performances of the simple physical structure model are presented as follows: the whole frame structure is made of an aluminum alloy with a Young’s modulus of E = 7300 MPa, a Poisson’s ration of v = 0.3, and a density of $\rho =2557\;\mathrm{kg}/m^{3}$. The weight of the whole frame structure is 43 kg (Figure 2b). The whole frame structure is composed of four supporting legs (the length of each leg is 1000 mm, and the diameter is 20 mm), a fixed platform (Figure 2c), a main reflector (Figure 2d), and a base (Figure 2e). The sub-reflector structure is composed of five iron plates. The weight of each iron plate (Figure 2f) is 7.5 kg and the total weight of the sub-reflector is 37.5 kg.

## 2. Construction of the Measurement Model

As shown in Figure 2, each supporting leg is regarded as a straight beam member of a constant circle cross-section. The inverse finite beam element quoted from References [15,17] is used to construct the measurement model. The process of constructing the measurement model is divided into four key steps: (1) the construction of the inverse finite beam element; (2) the computation of the section strains from the strain measurements; (3) the optimization of the strain sensor placement; (4) the construction of the measurement model in the global coordinate system.

### 2.1. Construction of the Inverse Finite Beam Element

This step is mainly quoted from References [15,17].

**Figure 3:**
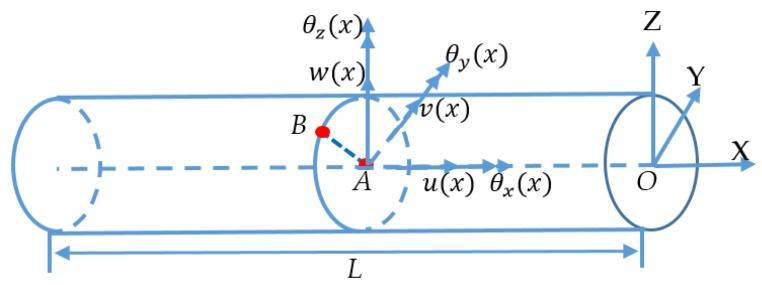
Beam geometry and kinematic variables.

Based on the assumption of Timoshenko theory, the displacement vector, which incorporates the three-dimensional Cartesian coordinates, is shown in Figure 3. The shift of a certain node, *B*, is presented as follows:

(1) $$\{\begin{array}{c} u_{x}\left(x,\;y,z\right)=u\left(x\right)+z\theta_{y}\left(x\right)-y\theta_{z}\left(x\right)\\ v_{y}\left(x,y,z\right)=v\left(x\right)-z\theta_{x}\left(x\right)\\ u_{z}\left(x,y,z\right)=w\left(x\right)+y\theta_{x}\left(x\right) \end{array}$$

where $\left\{u_{x}\left(x,y,z\right),\;v_{y}\left(x,y,z\right),u_{z}\left(x,y,z\right)\right\}$ indicates the shift of node B and (x,y,z) are the Cartesian coordinates of node B; $u\left(x,\;y,\;z\right)=\left\{u\left(x\right),v\left(x\right),w\left(x\right),\theta_{x}\left(x\right),\theta_{y}\left(x\right),\theta_{z}\left(x\right)\right\}$ represents the kinematic variables vector denoting the change of the node along the center axis (x∈[0,L],y=z=0), i.e. node A. In the finite element framework, the arbitrary-node kinematic variables vector u(x,y,z) is interpolated by the shape function N(x):

(2) $$u\left(x,\;y,z\right)=N\left(x\right)u^{e}$$

where $u^{e}$ denotes the nodal degrees-of-freedom. In light of Equation (2), the section strains vector $e\left(u^{e}\right)$ is obtained from the following equation:

(3) $$e\left(u^{e}\right)=B\left(x\right)u^{e}$$

where the matrix B(x) contains the derivatives of the shape function N(x). However, $e\left(u^{e}\right)$ cannot be directly obtained from the strain sensors. In iFEM, $e\left(u^{e}\right)$ is replaced with the in situ section strains vector $e^{\varepsilon }$ by minimizing the weighted least squares function φ:

(4) $$\varphi \left(u\right)=\|e\left(u\right)-e^{\varepsilon }{\|}^{2}$$

where $e^{\varepsilon }$ is computed from the surface strain measurements. Expanding the above least squares function results in the quadratic form:

(5) $$\varphi \left(u\right)=\frac{1}{2}{\left(u^{e}\right)}^{T}k^{e}u^{e}-{\left(u^{e}\right)}^{T}f^{e}+C^{e}$$

where $C^{e}$ is a constant vector; $k^{e}$ and $f^{e}$ are defined in the following:

(6) $$k_{k}^{e}=\frac{L}{n}\sum_{i=1}^{n}\left[B_{k}^{T}\left(x_{i}\right)B_{k}\left(x_{i}\right)\right]f_{k}^{e}=\frac{L}{n}\sum_{i=1}^{n}\left[B_{k}^{T}\left(x_{i}\right)e_{k}^{\varepsilon }\left(x_{i}\right)\right]$$

where L is the length of the beam element; n and $x_{i}$ $\left(0\leq x_{i}\leq L\right)$ are, respectively, the number and the axial coordinate of the locations where the section strains are calculated. In the following, the minimization of the function φ(u) in Equation (5) in terms of $u^{e}$ leads to the inverse finite beam element:

(7) $$k^{e}u^{e}=f^{e}$$

### 2.2. Computation of the Section Strains from the Strain Measurements

**Figure 4:**
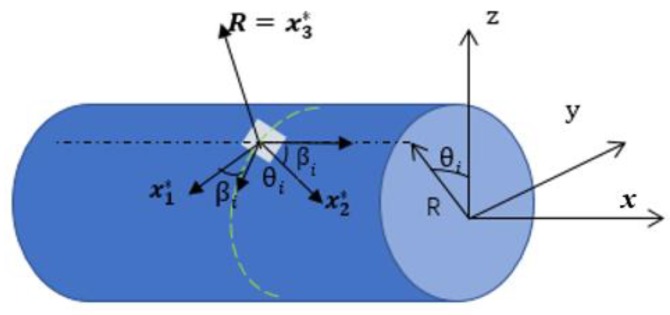
Location and coordinates of a strain sensor placed on an external beam surface [18].

The section strains are usually computed from the strain measurements through the following equation [15]:

(8) $$\begin{array}{cc} \varepsilon \left(x_{i},\theta_{i},\beta_{i}\right)= & e_{1}^{\varepsilon }\left(x_{i}\right)\left(c_{\beta }^{2}-vs_{\beta }^{2}\right)+e_{2}^{\varepsilon }\left(x_{i}\right)\left(c_{\beta }^{2}-vs_{\beta }^{2}\right)s_{\theta }R+e_{3}^{\varepsilon }\left(x_{i}\right)\left(c_{\beta }^{2}-vs_{\beta }^{2}\right)c_{\theta }R\\ & +e_{4}^{\varepsilon }\left(x_{i}\right)c_{\beta }s_{\beta }c_{\theta }-e_{5}^{\varepsilon }\left(x_{i}\right)c_{\beta }s_{\beta }s_{\theta }+e_{6}^{\varepsilon }\left(x_{i}\right)c_{\beta }s_{\beta }R\\ = & \left[c_{\beta }^{2}-vs_{\beta }^{2},\left(c_{\beta }^{2}-vs_{\beta }^{2}\right)s_{\theta }R,\left(c_{\beta }^{2}-vs_{\beta }^{2}\right)c_{\theta }R,c_{\beta }s_{\beta }c_{\theta },c_{\beta }s_{\beta }s_{\theta },c_{\beta }s_{\beta }R\right]\times e^{\varepsilon }\left(x_{i}\right)\\ = & T\left(x_{i},\theta_{i},\beta_{i}\right)\times e^{\varepsilon }\left(x_{i}\right)\\ & \mathrm{with}\;c_{\beta }\equiv \cos\beta_{i},s_{\beta }\equiv sin\beta_{i},c_{\theta }\equiv cos\theta_{i},s_{\theta }\equiv sin\theta_{i} \end{array}$$

where $e^{\varepsilon }\left(x_{i}\right)=\{e_{1}^{\varepsilon }\left(x_{i}\right),\;e_{2}^{\varepsilon }\left(x_{i}\right),\ldots ,\;e_{6}^{\varepsilon }\left(x_{i}\right)|\left(i=1,\;2,\;\ldots ,\;m\right)\}$ is the in situ section strains vector at location $x_{i}$ along the *x*-axis; m is the number of the sensors used to capture the surface strains of the beam; and *R* is the external radius of the beam element. $\varepsilon \left(x_{i},\;\theta_{i},\;\beta_{i}\right)$ denotes the measured strain at the location $\left(x_{i},\;\theta_{i},\;\beta_{i}\right)$, which is expressed in the cylindrical coordinate system (Figure 4). $T\left(x_{i},\;\theta_{i},\;\beta_{i}\right)$ is used to define the transformational relationship between the surface strain measurements and the section strains.

**Figure 5:**
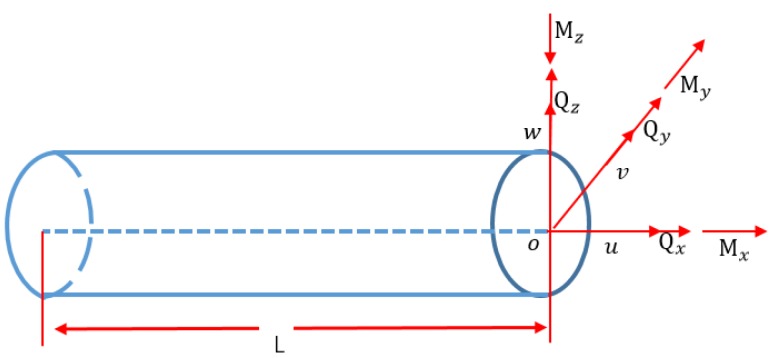
Beam section forces and moments [18].

When only Equation (8) is used, six strain sensors need to be placed in one section for the calculation of the section strains vector $e^{\varepsilon }$; thus, the total number of the strain sensors used to capture the surface strain measurements is 6 × *n* in one inverse finite beam element. Herein, *n* is the minimum number of the sections where the section strains are evaluated, which is different under different loading cases [21]. For the end-node load, *n* equals 2, whereas for the uniformly distributed load, *n* equals 3. With regard to the environmental loads, the deformations of four supporting legs mainly arise from the variations in gravity of the sub-reflector and the fixed platform during the pitching motion of the telescope. Also, the deformations of the four supporting legs are caused by the wind disturbance exerted on the surface of the sub-reflector. These loads are regarded as the end-node forces that are exerted on the four supporting legs. Therefore, the minimum number of sections is 2; the mini-number of the strain sensors is 12 in one inverse finite beam element. It is lucky that the number of the sensors can be reduced with the use of constitutive equations (Equation (9), refer to Figure 5) and equilibrium equations (Equation (10)).

(9) $$\begin{array}{ccc} N=A_{x}e_{1} & Q_{y}=G_{y}e_{5} & Q_{z}=G_{z}e_{4}\\ M_{x}=J_{x}e_{6} & M_{y}=D_{y}e_{2} & M_{z}=D_{z}e_{3} \end{array}$$

where the section forces ($N,\;Q_{y}\;and\;Q_{z}$) and the moments ($M_{x},\;M_{y}\;and\;M_{z}$) are related to the section strains $e_{i}\left(x\right)$ (*i* = 1, …,6). $A_{x}=EA$ is the axial rigidity, where A is the area of the cross-section of the beam element. $G_{y}=k_{y}^{2}GA$ and $G_{z}=k_{z}^{2}GA$ are the shear rigidities with $k_{y}^{2}$ and $k_{z}^{2}$ denoting the shear correction factors, where *G* is the shear modulus. For the solid section, $k_{y}^{2}$ = $k_{z}^{2}$ = 0.887. $J_{x}=GI_{p}$ is the torsional rigidity. $D_{y}=EI_{y}$. and $D_{z}=EI_{z}$. are the bending rigidities [15].

(10) $$\begin{array}{ccc} \frac{\partial N}{\partial x}+q_{x}=0 & \frac{\partial Q_{y}}{\partial x}+q_{y}=0 & \frac{\partial Q_{z}}{\partial x}+q_{z}=0\\ \frac{\partial M_{x}}{\partial x}=0 & \frac{\partial M_{y}}{\partial x}-Q_{z}=0 & \frac{\partial M_{z}}{\partial x}-Q_{y}=0 \end{array}$$

When the forms of the distribution loads $q_{x}$, $q_{y}$, and $q_{z}$ are known, the forms of the section strains can be estimated [15]. For the end-node loads, the section stains $e_{1}^{\varepsilon }\left(x_{i}\right)$, $e_{4}^{\varepsilon }\left(x_{i}\right)$, $e_{5}^{\varepsilon }\left(x_{i}\right)$, and $e_{6}^{\varepsilon }\left(x_{i}\right)$ are constant, while $e_{2}^{\varepsilon }\left(x_{i}\right)\;\mathrm{and}\;e_{3}^{\varepsilon }\left(x_{i}\right)$ are linear. In References [18,19], the section strains are expressed as:

(11) $$\begin{array}{lll} e_{1}^{\varepsilon }\left(x_{i}\right)=a_{1} & e_{2}^{\varepsilon }\left(x_{i}\right)=a_{2}x_{i}+a_{4} & e_{4}^{\varepsilon }\left(x_{i}\right)=m_{1}a_{2}\\ e_{6}^{\varepsilon }\left(x_{i}\right)=a_{6} & e_{3}^{\varepsilon }\left(x_{i}\right)=a_{3}x_{i}+a_{5} & e_{3}^{\varepsilon }\left(x_{i}\right)=a_{3}x_{i}+a_{5} \end{array}$$

or in a matrix equation:

(12) $$\begin{array}{cc} e^{\varepsilon }\left(x_{i}\right) & ={\left\{e_{1}^{\varepsilon }\left(x_{i}\right),e_{2}^{\varepsilon }\left(x_{i}\right),\ldots ,e_{6}^{\varepsilon }\left(x_{i}\right)\right\}}^{T}\\ & =[\begin{array}{cccccc} 1 & 0 & 0 & 0 & 0 & 0\\ 0 & x_{i} & 0 & 0 & 0 & 0\\ 0 & 0 & x_{i} & 0 & 1 & 0\\ 0 & m_{1} & 0 & 0 & 0 & 0\\ 0 & 0 & m_{1} & 0 & 0 & 0\\ 0 & 0 & 0 & 0 & 0 & 1 \end{array}]\times [a_{1},a_{2},a_{3},a_{4},a_{5},a_{6}{]}^{T}=T1(x_{i})\times p1,\\ & \mathrm{with}\;m_{1}=\frac{EI_{z}}{Gk_{y}^{2}A},\;m_{2}=\frac{EI_{y}}{Gk_{z}^{2}A} \end{array}$$

where $p1={\left[a_{1},\;a_{2},\;a_{3},\;a_{4},\;a_{5},\;a_{6}\right]}^{T}$ is a constant parameters vector; $T1_{x_{i}}$ defines the transfer matrix between p1 and the section strains vector $e^{\varepsilon }\left(x_{i}\right)$.

When the parameters vector p1 is solved, the arbitrary section strains vector along the *x*-axis is calculated through Equation (13).

(13) $$e^{\varepsilon }\left(x_{k}\right)=T1\left(x_{j}\right)\times {\left(T\left(x_{i},\theta_{i},\beta_{i}\right)T1\left(x_{i}\right)\right)}^{-1}\times \varepsilon \left(x_{i},\;\theta_{i},\;\beta_{i}\right)\;\left(i=1,\;\ldots ,\;6;\;k=1,\;2\right)$$

Finally, the kinematic variables vector $u^{e}$ is calculated through Equation (7) when two section strains vectors with respect to the two different cross-sections are determined. Subsequently, the deformation of an arbitrary node on the beam element surface is evaluated through Equation (1).

### 2.3. Optimization of the Strain Sensor Placement

**Figure 6:**
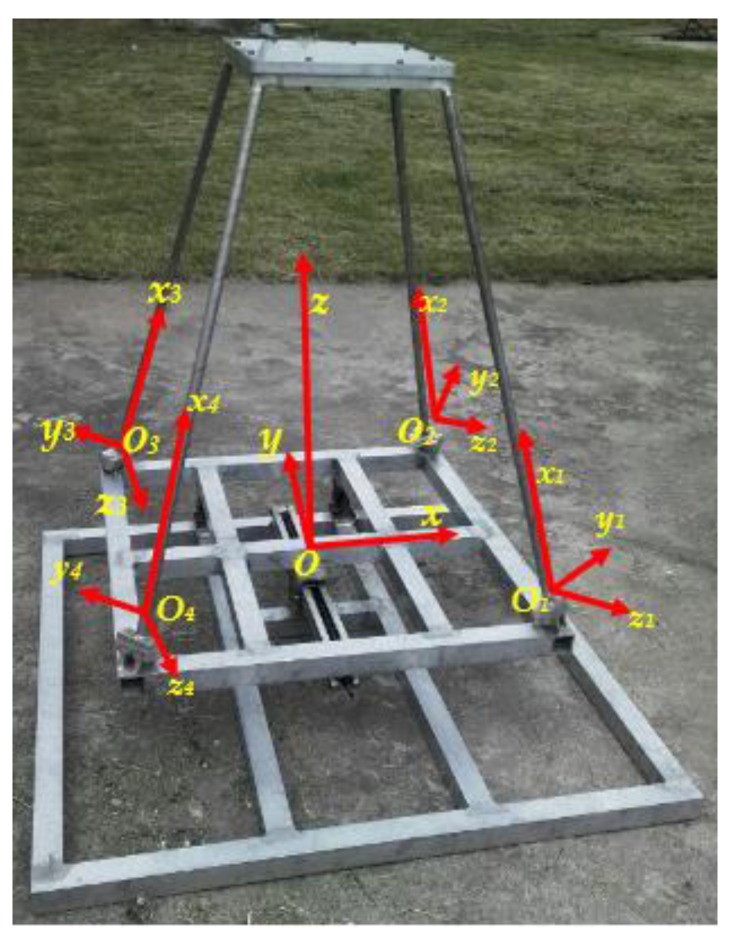
The coordinate system.

The estimation of the structural deformation from the strain measurements is ill-posed when the placement of strain sensors or the boundary condition is not appropriate [16]. For instance, when all the strain sensors are set parallel to the generatix of the beam (all the $\beta_{i}$ (*i* = 1, *…,m*) are set to the same value $0^{o}$ in Equation (13)), the transformation matrix $T\left(x_{i},\;\theta_{i},\;\beta_{i}\right)T1\left(x_{i}\right)$ in Equation (13) is singular, which causes the estimation of the beam structural deformation from the strain measurements by Equation (7) to be ill-posed. Zhao et. al, discussed the influence of the above ill-posed problem on the estimation of the beam structural deformation by Equation (7), and constructed an optimal placement model of strain sensors to maintain the stability and accuracy of the estimation of the beam structural deformation. Using the sensor placement scheme obtained from the aforementioned optimal placement model, the deformation of the beam/frame structure can be accurately and steadily reconstructed even if differences exist for strain sensor placement and strain measurement [18,19]. In this paper, the placement of strain sensors is quoted from References [18,19] (refer to Table 1). The coordinate of every sensor in Table 1 is in the coordinate system of Figure 4, which is a local coordinate system (refer to Figure 6).

### 2.4. Construction of the Measurement Model in a Global Coordinate System

As shown in Figure 3, Figure 4 and Figure 5, the coordinate system for the supporting leg reconstruction is a local coordinate system, where the *x*-axis is positioned along the centroidal axis of the beam element; *y* and *z* are the principal inertial axes of the cross-section. For the four supporting legs, there are four different local coordinate systems depicted in Figure 6, so it is necessary that these four local coordinate systems be unified into a global coordinate system. In this paper, the setup of the *x*-axis and the *y*-axis of the global coordinate system is conducted on the main reflector; the *z*-axis is perpendicular to the main reflector (Figure 6). 

The relationship between the local coordinate system of the *j^th^* supporting leg and the global coordinate system can be directly expressed as a direction cosine matrix:

(14) $$\Lambda_{j}={\left[\begin{array}{ccc} l_{x} & m_{x} & n_{x}\\ l_{y} & m_{y} & n_{y}\\ l_{z} & m_{z} & n_{z} \end{array}\right]}_{i}\;\left(j=1,2,3,4\right)$$

where $l_{k},\;m_{k},\;\mathrm{and}\;n_{k}$ (k=x, y, z) are the direction cosines of the local *k*-axis. Accordingly, the calculation model of the *j*^th^ supporting leg in the global coordinate system can be obtained as follows:

(15) $$\begin{array}{c} k_{j}^{\prime e}u_{j}^{\prime e}=f_{j}^{\prime e}\left(j=1,\;2,\;3,\;4\right)\\ \mathrm{with}\;k_{j}^{\prime e}=\Phi_{j}^{T}k_{j}^{e}\Phi_{j},\;f_{j}^{\prime e}=\Phi_{j}^{T}f_{j}^{e},\Phi_{j}=\left[\begin{array}{cc} \begin{array}{cc} \Lambda_{j} & 0\\ 0 & \Lambda_{j} \end{array} & \begin{array}{cc} 0 & 0\\ 0 & 0 \end{array}\\ \begin{array}{cc} 0 & 0\\ 0 & 0 \end{array} & \begin{array}{cc} \Lambda_{j} & 0\\ 0 & \Lambda_{j} \end{array} \end{array}\right] \end{array}$$

where $u_{j}^{\prime e}$ is used to denote the deformation of the *j*^th^ supporting leg in the global coordinate system.

## 3. Verification of the Model

**Figure 7:**
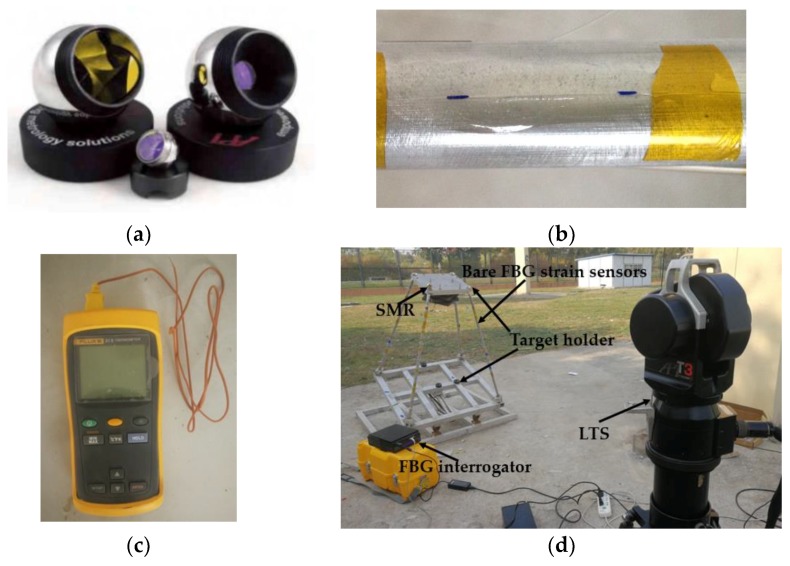
The test system of the simple physical structure: (**a**) Spherically Mounted Retro reflector (SMR) and target holder; (**b**) Bare FBG strain sensor attached to the beam surface; (**c**) Thermometer; (**d**) Test system.

In this section, a static loading test is conducted on a simple physical structure to verify the effectiveness and accuracy of the contact measurement scheme proposed in this paper. The real shifts of the fixed platform under different states are obtained using a laser tracking system, which comprises a Spherically Mounted Retro reflector (SMR, Figure 7a), seven different target holders (three target holders are installed on the main reflector to establish the principal coordinate system, while the other four are installed on the fixed platform to confirm the position and orientation of the fixed platform), and a laser tracking system (LTS, API Tracker 3, Automated Precision Inc., Rockwell, MD, USA). The resolution of the LTS is 1 µm and the accuracy is related to the distance between the LTS and the measured object (the ratio is 5 µm/m). In our test, the distance is 2 m, and the accuracy is 10 µm. The in situ strain is captured by the bare FBG strain sensor attached to the beam surface with alpha-cyanoacrylate glue (Figure 7b). To monitor the temperature variation, a thermometer (Figure 7c, 51 Series II, Fluke, Avery, WA, USA) is used to measure the temperature variation on the surface of the four supporting legs each half hour. In Reference [22], when the initial and current temperatures are known, the influence on the strain measurement can be estimated with Equation (16):

(16) $$\Delta \lambda_{i}=\left(\alpha +\eta \right)\Delta T_{j}\times \lambda_{ini\left(i\right)}$$

where $\Delta \lambda_{i}$ and $\lambda_{ini\left(i\right)}$ are, respectively, the wavelength variation affected by the temperature $\Delta T_{j}$ and the initial wavelength of the *i*^th^ FBG strain sensor attached to the surface of the *j*^th^ supporting leg. α is the thermal expansion of silica; η is the thermos-optic coefficient of the FBG sensor.

During the test, the in situ strain data are obtained from the strain measurement system, which comprises FBG strain sensors (Fiber Bragg Grating| os1100, Micron Optics, Atlanta, GA, USA) and an FBG interrogator (Optical Sensing Instrument| Si 155, Micron Optics, Atlanta, GA, USA). For each supporting leg, six FBG strain sensors (the range of the initial wavelength is (1527 nm, 1560 nm)) are placed on the surface of the beam according to the optimal placement scheme (see Table 1). Because four supporting legs possess the same performances, the placements of the strain sensors on four supporting legs are the same. Thus, the total number of the strain sensors used in the test is 24.

As mentioned in Section 1, the deformations of the four supporting legs give rise to the fixed platform shift. The real end-node shifts of the four supporting legs are computed using Equation (17):

(17) $${\left(\delta \right)}_{j}=\delta_{j}^{end}-\delta_{j}^{ini}\;(j=1,\;2,3,4)$$

where ${\left(\delta \right)}_{j}=\left(u_{j},v_{j},\;w_{j}\right)$ is the end-node (the intersection between the center axis of the supporting leg and the fixed platform) shift of the *j*^th^ supporting leg in the three-dimensional coordinate system. The superscript ‘*end*’ denotes the end-node coordinate in the current global coordinate system, whereas ‘ini’ denotes the initial end-node coordinate in the initial global coordinate system. 

**Figure 8:**
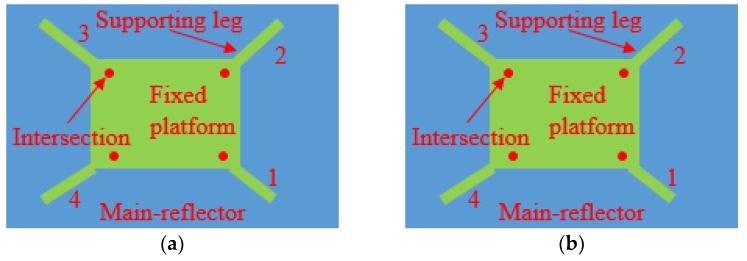
The top view of the fixed platform posture (the observation point is perpendicular to the main reflector): (**a**) The ideal posture; (**b**) The shift posture.

As shown in Figure 8, the fixed platform shift can be computed by using the shifts of the end-nodes of the four supporting legs through the following equations:

(18) $$LTS{\left(\delta \right)}_{o}=\sum_{j=1}^{4}LTS{\left(\delta \right)}_{j}/4,\;\;\mathrm{iFEM}{\left(\delta \right)}_{o}=\sum_{j=1}^{4}\mathrm{iFEM}{\left(\delta \right)}_{j}/4$$

where $\mathrm{iFEM}{\left(\delta \right)}_{j}\;\left(j=1,\;\ldots ,\;4\right)$ is the end-node shift of the *j*^th^ supporting leg computed from the in situ strain measurements with iFEM, while $LTS{\left(\delta \right)}_{j}$ is the end-node shift of the *j*^th^ supporting leg captured from the LTS. $\mathrm{iFEM}{\left(\delta \right)}_{o}$ is the shift of the fixed platform computed from $\mathrm{iFEM}{\left(\delta \right)}_{j}$, while $LTS{\left(\delta \right)}_{o}$ is the real shift of the fixed platform computed from $LTS{\left(\delta \right)}_{j}$.

**Figure 9:**
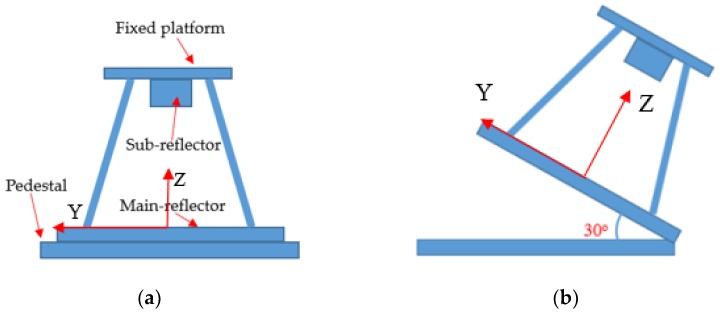

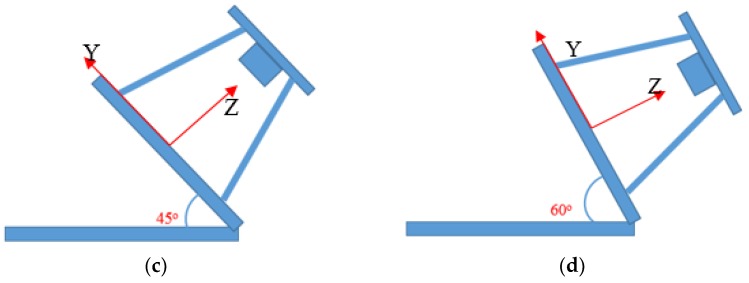
The side views of the supporting structure under different states: (**a**) Initial state; (**b**) Pitch angle: 30°; (**c**) Pitch angle: 45°; (**d**) Pitch angle: 60°.

The in situ strain measurements are captured from the bare FBG strain sensors. Different from traditional strain gauges such as resistance strain gauges, FBG sensors capture the structure strains based on the light wavelength shifts which are caused by FBG deformations due to the tension/compressive force or the temperature variation. In Reference [22], the strain is calculated through Equation (19) without considering the effect of temperature variation.

(19) $$\varepsilon_{i}=\frac{1}{K}*\left(\frac{\lambda_{end\left(i\right)}-\lambda_{ini\left(i\right)}-\Delta \lambda_{i}}{\lambda_{ini}}\right)\;with\;K=1-P_{e}$$

where $\lambda_{end\left(i\right)}$ and $\lambda_{ini\left(i\right)}$ are the current wavelength and the initial wavelength of the *i^th^* FBG sensor, respectively. $\Delta \lambda_{i}$ is the difference of the *i^th^* FBG sensor caused by the temperature variation, estimated using Equation (16). $P_{e}$ is the effective photo-elastic coefficient of the fiber; the strain measurement $\varepsilon_{i}$ is expressed as a micro strain. In the test, the wavelength of the FBG sensor is defined as the initial wavelength at the initial state of the supporting structure (Figure 9a), whereas the wavelength of the FBG sensor is defined as the current wavelength when the supporting structure is leaning at a certain angle (Figure 9b–d). The typical strain measurements at the pitch angle 30° are shown in Table 2.

Table 3 presents the corresponding real shifts of the fixed platform ($LTS{\left(\delta \right)}_{o}$) measured from the LTS and the evaluated shifts of the fixed platform ($\mathrm{iFEM}{\left(\delta \right)}_{o}$) computed from the in situ strain measurements with iFEM. Also, the accuracies of the evaluated shifts are assessed by means of the absolute error and the percent difference:

(20) $$\begin{array}{c} \mathrm{Diff}{\left(\delta \right)}_{o}=\left|\mathrm{iFEM}{\left(\delta \right)}_{o}-API{\left(\delta \right)}_{o}\right|\\ \%\mathrm{Diff}{\left(\delta \right)}_{o}=100\times \left[\frac{\left|\mathrm{iFEM}{\left(\delta \right)}_{o}-API{\left(\delta \right)}_{o}\right|}{\left|API{\left(\delta \right)}_{o}\right|}\right] \end{array}$$

Furthermore, the orientation of the fixed platform can be calculated from the shift of the fixed platform and the shifts of the intersections (see Figure 8) of the four supporting legs using a discrete cosine algorithm (see Table 4).

From Table 3, it was found that the shifts of the fixed platform can be accurately evaluated from the strain measurements with iFEM at different pitch angles. Specifically, the percent differences of the shifts between iFEM calculations and LTS captures are below 6% along the maximum-shift direction (*y*-direction). Although the percent differences of the shifts along the other two directions (*x*- and *z*- directions) are bigger than those along the *y*-direction, the absolute shifts along the *x*- and *z*- directions are below 0.1 mm, smaller than those along the *y*-direction. Moreover, the absolute differences between iFEM calculations and LTS captures are less than or equal to 0.08 mm. From Table 4, it was found that the percent differences of the orientations are below 1% for *x*-rotation and *z*-rotation. Although the percent differences of the orientations for *y*-rotation are bigger than those for *x*-rotation and *z*-rotation, the absolute differences of the orientations for *y*-rotation are below 0.02°. 

## 4. Conclusions

With aim of providing the exact adjustment values for the sub-reflector adjustment structure of a large radio telescope, a contact measurement scheme was proposed to monitor the position and orientation of the sub-reflector in real time. In the scheme, several FBG strain sensors are attached to the surfaces of the four supporting legs and the deformations of these supporting legs are computed from in situ strain measurements to evaluate the position and orientation of the fixed platform. In order to verify the accuracy and effectiveness of the contact measurement scheme, a static test was conducted on a simple physical structure. The experimental results demonstrate that the contact measurement scheme proposed in this paper can effectively and accurately evaluate the shift of the fixed platform, offering a new way to monitor the shift of a radio telescope sub-reflector. 

## Figures and Tables

**Table 1:** Description of the optimal placement for six strain sensors; angles are expressed in degrees.

**Description**	Orientation (θ,β) $\mathrm{at}\;x_{i}=L/5$	Orientation (θ,β) $\mathrm{at}\;x_{i}=4L/5$
**Six Strain Sensors**	(−120,0), (0,0), (120,0)	(−120,0), (0,45), (120,0)

**Table 2:** The typical strain measurements at a pitch angle of 30°.

Sensor Location	Supporting Leg 1	Supporting Leg 2	Supporting Leg 3	Supporting Leg 4
L/5, −120°,0°	0.000079	0.000112	−0.000172	−0.000166
L/5, 0°,45°	0.000019	0.000028	0.000049	0.000053
L/5, 120°,0°	−0.000272	−0.00019	0.000132	0.000107
4L/5, −120°,0°	−0.000335	−0.000295	0.000263	0.000261
4L/5, 0°,45°	−0.000024	−0.000015	0.000001	−0.000046
4L/5, 120°,0°	0.000429	0.000327	−0.000395	−0.000289

**Table 3:** The shifts measured using API Tracker 3 and the shifts computed from the strain data using the inverse Finite Element Method (iFEM).

Pitch Angle		Shift in *x-*Direction	Shift in *y*-Direction	Shift in *z-*Direction
30°	$LTS{\left(\delta \right)}_{o}$	−0.01 mm	−0.62 mm	0.08 mm
	$\mathrm{iFEM}{\left(\delta \right)}_{o}$	0.03 mm	−0.59 mm	0.0 mm
	$\mathrm{Diff}{\left(\delta \right)}_{o}$	0.04 mm	0.03 mm	0.08 mm
	$\%\mathrm{Diff}{\left(\delta \right)}_{o}$	400%	4.8%	100%
45°	$LTS{\left(\delta \right)}_{o}$	−0.02 mm	−1.82 mm	0.03 mm
	$\mathrm{iFEM}{\left(\delta \right)}_{o}$	0.05 mm	−1.73 mm	0.01 mm
	$\mathrm{Diff}{\left(\delta \right)}_{o}$	0.07 mm	0.09 mm	0.02 mm
	$\%\mathrm{Diff}{\left(\delta \right)}_{o}$	350%	4.9%	66.7%
60°	$LTS{\left(\delta \right)}_{o}$	0.03 mm	−2.06 mm	−0.04 mm
	$\mathrm{iFEM}{\left(\delta \right)}_{o}$	0.08 mm	−1.95 mm	−0.04 mm
	$\mathrm{Diff}{\left(\delta \right)}_{o}$	0.05 mm	0.11 mm	0.0 mm
	$\%\mathrm{Diff}{\left(\delta \right)}_{o}$	166.7%	5.3%	0.0%

**Table 4:** The comparison of the fixed platform orientations at different pitch angles.

Pitch Angle		*x*-Rotation	*y*-Rotation	*z*-Rotation
30°	LTS(θ)	1.626°	−0.02°	0.0°
	iFEM(θ)	1.619°	−0.013°	0.0°
	Diff(θ)	0.007°	0.007°	0.0°
	%Diff(θ)	0.4%	35%	0.0%
45°	LTS(θ)	1.722°	−0.04°	0.21°
	iFEM(θ)	1.709°	−0.027°	0.226°
	Diff(θ)	0.013°	0.013°	0.016°
	%Diff(θ)	0.8%	32.5%	1.9%
60°	LTS(θ)	1.721°	−0.066°	0.5°
	iFEM(θ)	1.704°	−0.048°	0.504°
	Diff(θ)	0.017°	0.018°	0.004°
	%Diff(θ)	0.98%	27.3%	0.8%

**Figure A1:**
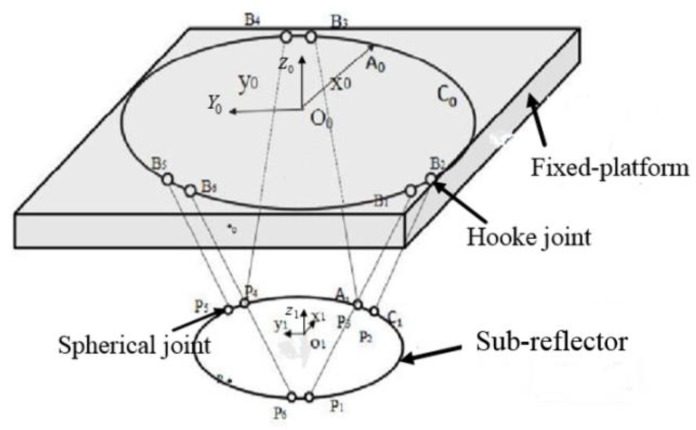
The coordinate systems of the fixed platform and the sub-reflector.

## Appendix A

$U_{0},\;V_{0}$ are, respectively, defined as the initial position and initial orientation of the sub-reflector; the corresponding changes are ΔU and ΔV in the case of pitching. Thus, the new position and orientation of the sub-reflector are:

(A1) $$U_{1}=R_{1}U_{0}+\Delta U,\;V_{1}=V_{0}-\Delta V$$

where $R_{1}$ is used to define the transform matrix between the initial state and the current state of the fixed platform.

In order to maintain the original position and orientation of the sub-reflector, the length of the Stewart platform leg will change through Equation (A2):

(A2) $$\Delta L_{i}=\|R_{2}P_{i}+U_{1}-B_{i}\|-\|R_{3}P_{i}+U_{0}-B_{i}\|\;i=1,2,\ldots ,6$$

where $R_{2}$ is the transform matrix between the initial state of the sub-reflector and the current state of the fixed platform. $R_{3}$ is the transform matrix between the fixed platform and the sub-reflector under the initial state. $P_{i}$ and $B_{i}$ are used to define the coordinates of the *i*^th^ spherical joint and the coordinate of the *i*^th^ Hooke joint, respectively (Figure A1).
